# Mouse Model of Cat Allergic Rhinitis and Intranasal Liposome-Adjuvanted Refined Fel d 1 Vaccine

**DOI:** 10.1371/journal.pone.0150463

**Published:** 2016-03-08

**Authors:** Natt Tasaniyananda, Urai Chaisri, Anchalee Tungtrongchitr, Wanpen Chaicumpa, Nitat Sookrung

**Affiliations:** 1 Graduate Program in Immunology, Department of Immunology, Faculty of Medicine Siriraj Hospital, Mahidol University, Bangkok 10700, Thailand; 2 Department of Parasitology, Faculty of Medicine Siriraj Hospital, Mahidol University, Bangkok 10700, Thailand; 3 Department of Tropical Pathology, Faculty of Tropical Medicine, Mahidol University, Bangkok 10400, Thailand; 4 Department of Research and Development, Faculty of Medicine Siriraj Hospital, Mahidol University, Bangkok 10700, Thailand; Chang-Gung University, TAIWAN

## Abstract

Cats (*Felis domesticus*) are rich source of airborne allergens that prevailed in the environment and sensitized a number of people to allergy. In this study, a mouse model of allergic rhinitis caused by the cat allergens was developed for the first time and the model was used for testing therapeutic efficacy of a novel intranasal liposome-entrapped vaccines made of native Fel d 1 (major cat allergen) in comparison with the vaccine made of crude cat hair extract (cCE). BALB/c mice were sensitized with cCE mixed with alum intraperitoneally and intranasally. The allergic mice were treated with eight doses of either liposome (L)-entrapped native Fel d 1 (L-nFD1), L-cCE), or placebo on every alternate day. Vaccine efficacy evaluation was performed one day after provoking the treated mice with aerosolic cCE. All allergenized mice developed histological features of allergic rhinitis with rises of serum specific-IgE and Th2 cytokine gene expression. Serum IgE and intranasal mucus production of allergic mice reduced significantly after vaccination in comparison with the placebo mice. The vaccines also caused a shift of the Th2 response (reduction of Th2 cytokine expressions) towards the non-pathogenic responses: Th1 (down-regulation of the Th1 suppressive cytokine gene, *IL-35*) and Treg (up-regulation of *IL-10* and *TGF-β*). In conclusions, a mouse model of allergic rhinitis to cat allergens was successfully developed. The intranasal, liposome-adjuvanted vaccines, especially the refined single allergen formulation, assuaged the allergic manifestations in the modeled mice. The prototype vaccine is worthwhile testing further for clinical use in the pet allergic patients.

## Introduction

Cats contribute a rich source of airborne allergens that sensitize about 5–20% of atopic patients [1,2]. Clinical manifestations of the cat allergy include chronic allergic rhinitis (AR) and asthma which impair the patient’s capacity and increase economic burden. The cat allergens may be found in places without cats or they remain for many months after the cats were taken away and the places were regularly cleaned [3,4]. Therefore avoiding cats for morbidity intervention is a difficult practice for the cat allergic subjects. Among the known cat allergens, Fel d 1, which is mainly found in the cats’ hair, dander and/or saliva [4] is the most potent allergen as it binds to serum IgE of up to 90% of the cat allergic subjects [5]. Currently, allergen-specific immunotherapy (SIT) is the only disease modifying/curative treatment option of allergy [6]. To do so, the patient is given increasing amounts of the allergen either parenterally (e.g., subcutaneous/intradermal) or mucosally (e.g., sublingual), over an extended period of time until the maintenance dose is reached. The maintenance doses are then given further for many more years [7]. The aim is to cause a deviation from the pathogenic Th2 towards the non-pathogenic Th1 and/or regulatory T cell (Treg) responses. However, the SIT receives low patients’compliance as not only it is time-consuming and prolonged, but also confers a possible risk of adverse reactions, *e*.*g*., life-threatening anaphylaxis.

In this study, a mouse model of allergic rhinitis to cat allergens was developed for testing efficacies of intranasal liposome-entrapped vaccines made of crude cat hair extract (cCE) or refined Fel d 1. Liposome is a safe vaccine delivery vehicle and promising immunological adjuvant [8–10]. The intranasal route is non-invasive and relatively immunogen sparing compared to the sublingual immunization. The immune responses can be expected from the local lymphoid tissues which should be effective locally [9]. The native Fel d 1 was used as a vaccine component as evidences suggested that refined allergen is better than the crude extract in reducing the allergic immune responses [9,11,12]. The refined allergen is easy to standardize and also free of other unidentified and non-allergenic components.

## Materials and Methods

### Reagents

CNBr-activated Sepharose 4B resin was from GE Healthcare, UK; didodecyldimethylammonium bromide (DDAB) was from Fluka, Germany; phosphatidylcholine (soybean lecithin, Lipoid-S-100) was from Lipoid AG, Switzerland. RNA*later* RNA stabilization reagent (RNA *later*^TM^) was from QIAGEN GmbH, Hilden, Germany; Phusion Hot Start II DNA Polymerase, Anchored Oligo dT, RevertAid First Strand cDNA Synthesis Kit, HisPur^TM^ Ni-NTA Resin and Imject^TM^ Alum Adjuvant were from Thermo Fisher Scientific, MA, USA; Isopropyl-β-D-Thiogalactopyranoside (IPTG) was from affymetrix, USB, CA, USA; Total RNA Mini Kit from Geneaid Biotech, Taiwan; cholesterol, dichloromethane, paraformaldehyde and Tween-20 were from Sigma-Aldrich, Germany.

### Preparation of crude cat hair extract, and native and recombinant Fel d 1

Each gram of the hair of healthy cats was added with 20 ml PBS containing 0.05% Tween-20 (PBST), sonicated (40 kHz) at 4–8°C for 30 min, filtered through a cell strainer, and centrifuged at 2,000 ×*g*, 4°C for 30 min. The supernatant was dialyzed against distilled water at 4°C. Protein content of the cCE was determined.

Native Fel d 1 (nFel d 1) was purified from the cCE by mouse monoclonal antibody (mAb) based-affinity resin. Fel d 1-specific mouse mAb was added to the CNBr-activated Sepharose 4B resin (GE Healthcare, UK) and the preparation was rotated at 25°C for 1 h. Excess antibody was removed; the resin was washed with the coupling buffer and blocked with 0.1 M Tris-HCl, pH 8.0, for 2 h. After washing several times with 0.1 M acetic acid/sodium acetate, pH 4.0 containing 0.5 M NaCl followed by 0.1 M Tris-HCl, pH 8.0 containing 0.5 M NaCl, cCE was mixed with the mAb-adsorbed resin and rotated at 25°C for 2 h. After washing, the resin was packed into a 15 × 80 mm column (PD-10, GE Healthcare). Native Fel d 1 was eluted out using 0.1 M glycine-HCl, pH 2.5, neutralized immediately with 1 M Tris-HCl, pH 8.0, and dialyzed against PBS before concentrating to 5 mL.

Recombinant Fel d 1 (rFel d 1) was prepared from a transformed *E*. *coli* carrying *Fel d 1*-plasmids [13]. The *E*. *coli* cells grown under 0.4 mM IPTG induction were sonicated in lysis buffer (4% glycerol in 10 mM Tris-HCl, pH 7.4) and centrifuged at 15,000 × *g* for 20 min. The rFel d 1 was purified from the bacterial lysate by using HisPur^TM^ Ni-NTA Resin (Thermo Scientific, USA).

### Cat allergy (allergic rhinitis) model

Animal experiments were approved by Animal Care and Use Committee, Faculty of Medicine Siriraj Hospital (SiACUC), Mahidol University (COA No. 011/2558). Female BALB/c mice, 6–8 weeks old from the National Laboratory Animal Center, Mahidol University, were sensitized intraperitoneally with three doses of cCE containing 10 μg of nFel d 1 in PBS mixed (2:1 v/v) with alum adjuvant (Thermo Scientific) (total volume 200 μL) on days 0, 7 and 14. On days 21–27, each mouse was challenged daily and intranasally (i.n.) with 20 μl of cCE in PBS containing 1 μg of Fel d 1 (10 μL per nostril). On days 34, 35 and 36, mice were nebulized with 10 mg of cCE in 10 mL PBS. Sham mice received PBS instead of the cCE. One day 37, all mice were bled and sera were collected. Some mice were sacrificed for monitoring allergic status. S1 Fig shows timeline for cat allergy model development.

### Liposome and vaccine formulations

Multi-lamellar liposome was prepared and used as the vaccine/placebo delivery vehicle [9,14]. Briefly, 153 mg of DDAB (Fluka, Germany), 148 mg of phosphatidylcholine (soybean lecithin, Lipoid-S-100, Lipoid AG, Switzerland) and 72.5 mg of cholesterol (Sigma-Aldrich, Germany) were mixed (molar ratio 2:1:1) using dichloromethane as a solvent. One ml of the lipid stock was rotated in a round bottom-flask until a thin film was obtained.

Two vaccine formulations were prepared: liposome entrapped cCE (L-cCE) and liposome entrapped nFel d 1 (L-nFD1). For L-cCE, 1.67 mg of cCE (containing 150 μg of Fel d 1) in 500 μL PBS were added to the lipid film prepared from 1 ml of the lipid stock solution and mixed until a milky homogeneous suspension was obtained. For L-nFD1, nFel d 1 (150 μg) in 500 μL PBS was added to the lipid film. Liposome entrapped PBS (L-P) was prepared similarly. Polydispersity indices (PDI) and zeta-potentials of the liposome particles were measured by dynamic light scattering and electrophoresis technique, respectively, using a particle size analyzer (Zetasizer Nano ZS, Malvern Instrument Limited, UK). The percentage of the immunogen entrapment was determined [9].

### Mouse vaccination and provocation and vaccine efficacy evaluation

Two weeks after the cCE nebulization, the remaining allergic mice were divided into 3 groups. Group 1 (placebo) mice were given L-P (20 μL) i.n. Groups 2 and 3 were treated i.n. with 20 μL of L-cCE (containing 66 μg of cCE) and L-nFD1 containing 6 μg of nFel d 1, respectively. Seven booster doses were given on every alternate day. One week after the last booster (day 71), mice were provoked with 10 mg of cCE in 10 mL PBS using nebulizer. S2 Fig shows timeline for mouse vaccination, provocation and vaccine efficacy evaluation.

Immediately after provocation, frequencies of nose rubbing and sneezing of all mice were recorded by a person who was blinded of the mouse treatments during the following 15 min. Mice were bled on day 72 (one day post-provocation) and serum samples were collected for measuring the levels of specific Fel d 1 antibodies. Thereafter, mice were sacrificed. The mouse nasal tissues were used for cytokine gene expressions and histopathology.

### Indirect ELISA

Levels of rFel d 1-specific IgE, IgG1 and IgG2a in mouse sera were determined by indirect ELISA [9]. Individual sera were diluted 1:10 for IgE and 1:1,000 for IgG1 and IgG2a determination. Mice with specific IgE higher than mean + 2 SD of the sham sera were regarded as allergic mice.

### Histopathological study

For histopatholical study, right side of each mouse head was fixed in 5% paraformaldehyde and 4% sucrose in PBS. Five μm tissue sections were prepared and they were stained either with hematoxylin and eosin dyes (H & E) for neutrophil, lymphocyte and eosinophil; toluidine blue dye for mast cells; and Periodic acid-Schiff (PAS) reagent for mucus. All stained sections were observed under a light microscope (400×) (BX41, Olympus,Tokyo, Japan) with DP2-BSW software by a pathologist who was blinded on the mouse treatment groups. The cells along the epithelium in at least 10 microscopic fields per section per mouse were counted. PAS-stained mucus glands in the tissues were graded arbitrarily based on the intensity of the tissue color (magenta) by using scales 1–3. Percentages of individual mucus stained grades were calculated from a total number of the microscopic fields of each grade divided by a total number of the inspected fields in each group ×100.

### Cytokine study

Quantitative real-time PCR (qPCR) was used for monitoring cytokine gene expression. Left side of the mouse head was put in RNA*later* RNA stabilization reagent (RNA *later*^TM^, QIAGEN GmbH, Hilden, Germany). Total RNA was extracted from the soft nasal tissues by using Total RNA Mini Kit (Geneaid Biotech, Taiwan). Complementary DNA (cDNA) was synthesized (SuperScript®III CellsDirect cDNA synthesis system; Invitrogen^TM^, Life Technologies, Thermo Fisher Scientific, USA). Cytokine mRNAs including *IL-4*, *IL-5*, *IL-13*, *TNF-α*, *IL-12a (p35)*, *IL-12b (p40)*, *IL-23 (p19)*, *IFN-γ*, *IL-10*, *TGF-β*, and *IL-35* (*ebi3*) were quantified using the cDNA as templates and *β-actin* mRNA for normalization. The nucleotide primers [15–22] are listed in S1 Table. The PCR mixture contained 1 μL of cDNA and 100 nM of each primer in a SYBR Green PCR Master Mix (Applied Biosystems, USA). MxPro QPCR software for Mx3005P QPCR System (Agilent Technologies, USA) was used for data analysis.

### Statistical analyses

SPSS 17.0 statistical software was used. One-way ANOVA, post hoc comparison using least significant difference (LSD) and independent*-t*-test were applied for analyses of antibody levels and histopathological and cytokine data. Percentages of mucus grades were compared by Chi-square test. *P* <0.05 was significantly different.

## Results

### Allergy model

Frequencies of nose rubbing and sneezing of the allergenized mice after aerosolic cCE challenge were significantly higher than those of the sham group (*p* < 0.05).

Normal and sham mice did not have detectable serum specific IgE, IgG1 and IgG2a to rFel d 1 at the serum dilutions used in the indirect ELISA (1:10 for IgE and 1:1000 for IgG1 and IgG2). The means ± SDs of the OD_405nm_ of specific IgE, IgG1 and IgG2a in sera of allergenized mice were 0.582 ± 0.273, 1.212 ± 0.152, and 0.051 ± 0.057, respectively. Based on their serum specific IgE levels, all sensitized mice were allergic to the cCE.

**Fig 1:**
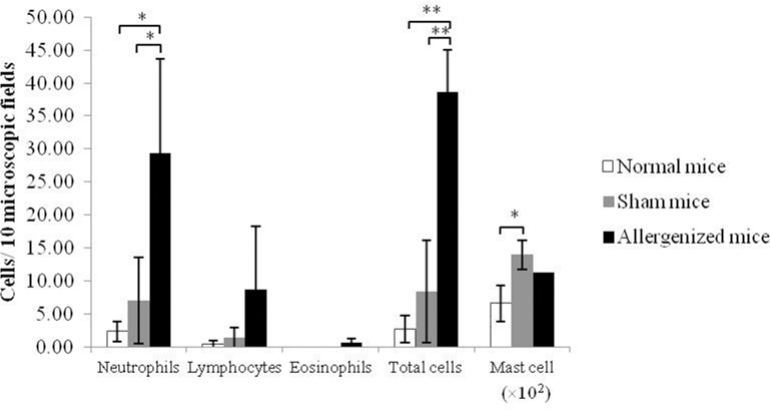
Mean ± SD numbers of inflammatory cells in nasal tissues of normal, sham, and allergenized mice. ^*^ *P* < 0.05, ^**^ *P* < 0.01.

Normal and sham mice had fewer neutrophils, lymphocytes, and eosinophils than the allergenized mice. The number of combined neutrophils, lymphocytes, and eosinophils in the nasal tissues of cCE-allergenized mice was significantly higher than those of normal and sham mice (*p* < 0.05) (Fig 1). Mast cells were predominant at the tip of the mouse nose, and almost negligible in the nasal tissue elsewhere. Allergenized and sham mice had many more mast cells than the normal mice (*p* < 0.05) (Fig 1).

**Fig 2:**
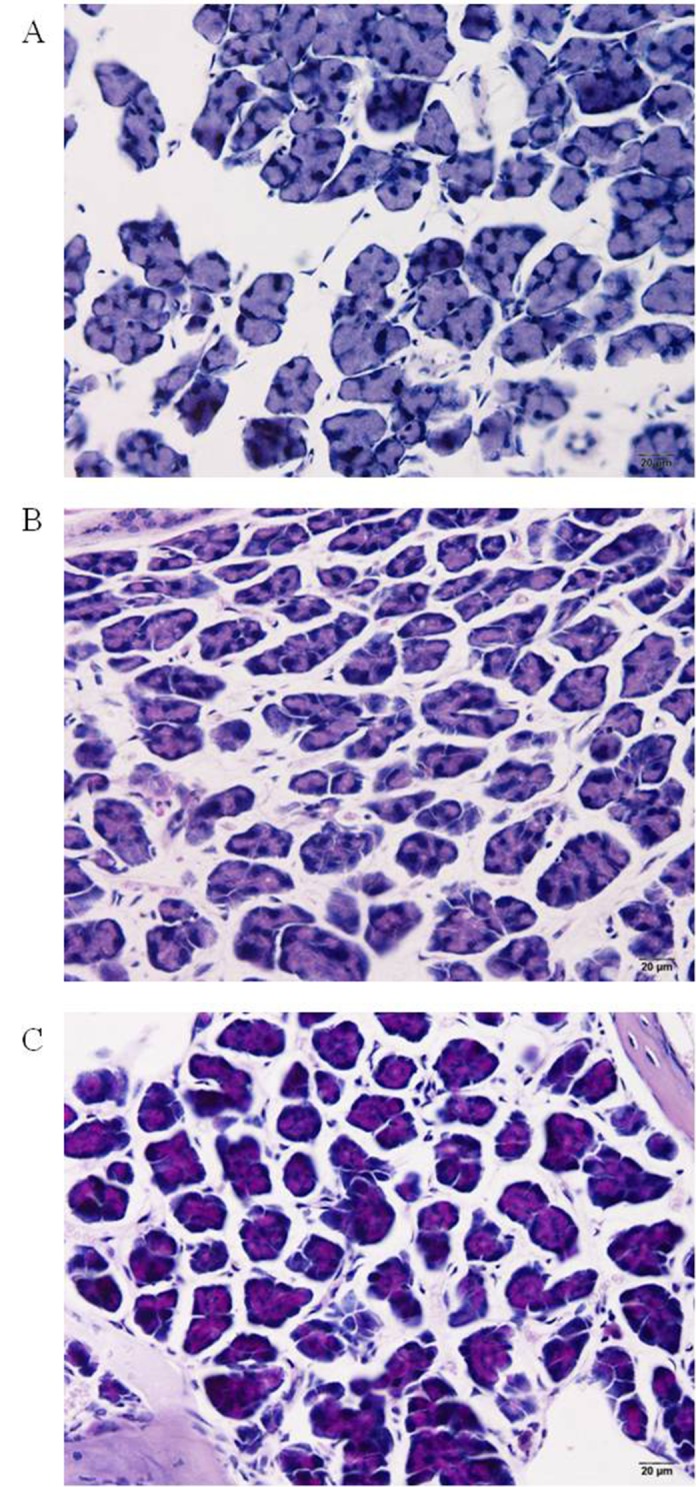
(A-C) Grades 1–3 of color intensity of the PAS-stained mucus glands in nasal tissues of mice (original magnification 400×). The color (magenta) is a result of the reaction between PAS dye and glycogens in the mucus.

Fig 2 shows intensity of grades 1–3 of PAS-stained mucus glands in the mouse nasal tissues. Percentages of individual grades of the PAS-stained mucus in nasal tissues of normal, allergenized, and sham mice are shown in Table 1. The grade 3 mucus gland intensity was found only in the cCE-allergenized mice indicating that these mice had more active mucus glands than the normal and sham mice.

**Table 1:** Percentages of the PAS Stained Mucus Grades in Nasal Tissues of Normal, Sham and Allergenized Mice and Allergic Mice after Receiving Vaccines and Placebo.

Group of mice (Total microscopic fields)	% mucus grade^*^		
	Grade 1	Grade 2	Grade 3
Normal (31)	83.87^a^	16.13^a^	0^a^
Cat CE allergenized (23)	56.52^b^	26.09^a^	17.39^b^
Sham (53)	81.13^a^	18.87^a^	0^a^
L-P (78)	48.71^a^	23.08^a^	28.21^a^
L-cCE (92)	55.44^a^	42.39^b^	2.17^b^
L-nFD1 (55)	47.27^a^	40.00^b^	12.73^b^

L-P, L-cCE and L-nFD1 are allergic mice after receiving placebo and vaccines (5 mice per group). Five to twenty microscopic fields (400×) of the mucus glands in the stained nasal tissues were graded according to the color intensities.

^*^ Percentages of individual PAS stained mucus grades were analyzed by Chi-square test.

Entries with different superscripts along vertical axis (a *versus* b) are statistically different at *p* < 0.05.

### Characteristics of liposome and liposome entrapped vaccines/placebo

The sizes, zeta potentials, PDI and percentages of immunogen entrapment of the two vaccine formulations and the placebo (L-P) are shown in Table 2. Sizes of the liposome of all formulations were 3.5–5.4 μm with small PDI, indicating high homogeneity of the vesicles. The L-cCE had a slightly anionic charge while the L-nFD1 and L-P were carrying cathodic surface charge. The average percentages of the immunogen entrapment were 74.63 and 73.48% for L-cCE and L-nFD1, respectively.

**Table 2:** Characteristics of the Liposome Entrapped Vaccines and Placebo.

	Vaccine		Placebo
Parameter	L-cCE	L-nFD1	L-P
Average size (nm) (Mean ± SD)	5,345.33 ± 170.9	3,526.33 ± 284.01	4,519.33 ± 191.11
Polydispersity index (PDI) (Mean ± SD)	0.244 ± 0.11	0.535 ± 0.06	0.489 ± 0.003
Zeta potential (mV) (Mean ± SD)	-2.31 ± 0.25	+41.40 ± 0.0	+26.73 ± 0.12
% immunogen entrapment	74.63	73.48	N/A

N/A, not applicable

### Symptom scores, inflammatory cells and mucus in nasal tissues and serum specific antibodies of vaccinated/placebo mice

Frequencies of sneezing and nose rubbing among the vaccinated and placebo mice were not different during the 15 min post-provocation.

The average numbers of inflammatory cells of placebo/vaccinated mice in nasal epithelia were not different after placebo/vaccine treatments and provocation (*p* > 0.05).

There was a significant reduction of the grade 3 mucus glands in both L-cCE and L-nFD1 treated mice. On contrary, the placebo mice had a percent increment of the grade 3 mucus glands (Table 1).

**Fig 3:**
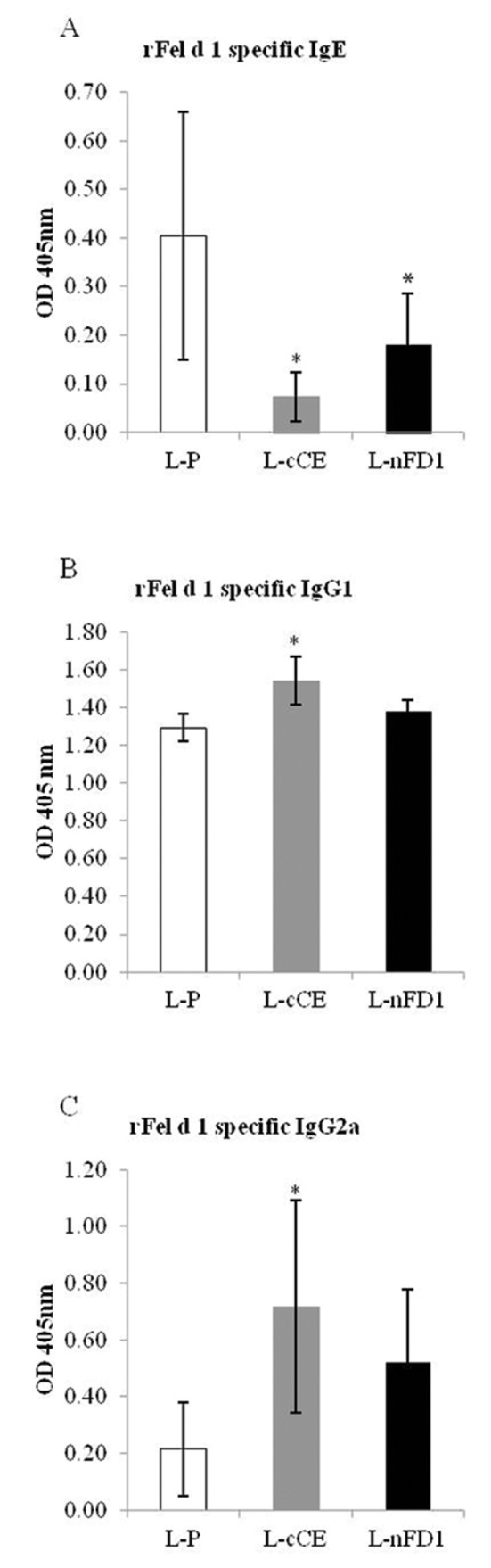
Means of indirect ELISA OD_405nm_ of rFel d 1 specific serum (A) IgE, (B) IgG1 and (C) IgG2a of cCE allergic mice after receiving vaccines/placebo + provocation with aerosolic cCE. ^*^ *P* < 0.05 compared with L-P by independent *t*-test.

Specific IgE, IgG1 and IgG2a to Fel d 1 of the cCE allergic mice after receiving vaccines/placebo + provocation are shown in Fig 3. The mean IgE levels of L-cCE and L-nFD 1 vaccinated allergic mouse groups were not different (*p* > 0.05) and both were lower significantly than of the placebo (*p* < 0.05) and was not different from the L-nFD1 group Allergic mice that received L-cCE treatment had significant increase of the allergen specific IgG1 and IgG2 when compared to placebo (*p* < 0.05). Specific IgG1 and IgG2 levels in the L-nFD1 vaccinated allergic mice were not different from the placebo mice (*p* > 0.05). Nevertheless, when the IgE:IgG1 and IgE:IgG2 ratios of all groups were worked out, it was found that both vaccinated mouse groups had significantly less values of the antibody ratios compared to the placebo mice (S2 Table).

### Cytokine genes expressions

**Fig 4:**
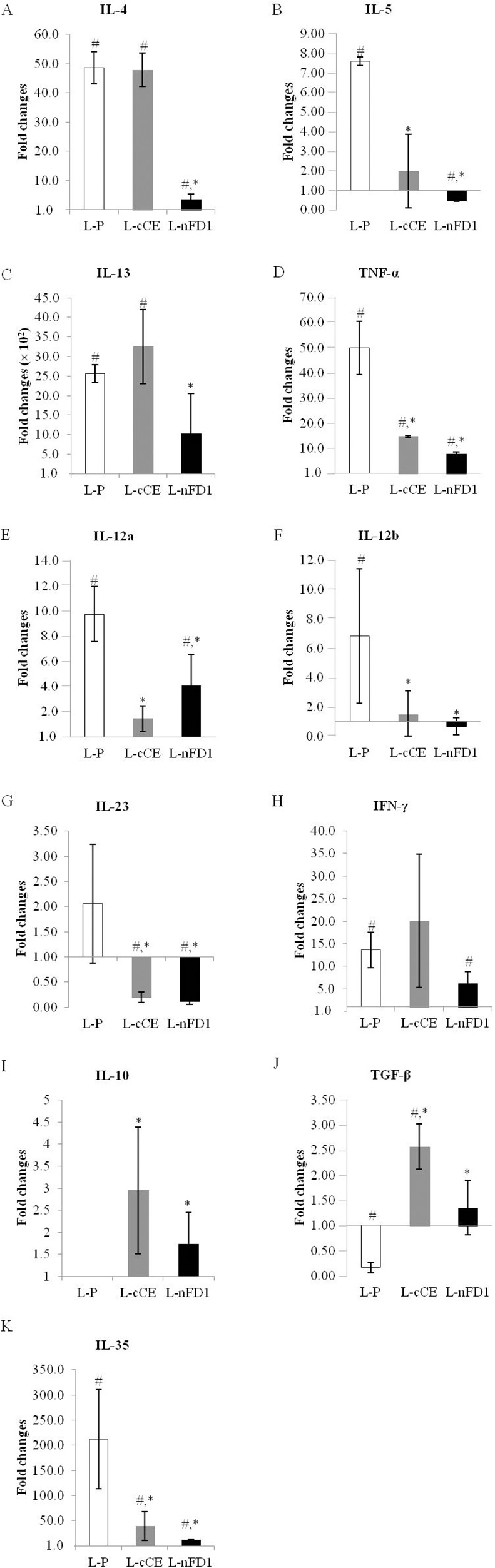
Fold changes of cytokine mRNAs (A) *IL-4*, (B) *IL-5*, (C) *IL-13*, (D) *TNF-α*, (E) *IL-12a (p35)*, (F) *IL-12b (p40)*, (G*) IL-23 (p19)*, (H) *IFN-γ*, (I) *IL-10*, (J) *TGF-β* and (K) *IL-35* in nasal tissues of allergic mice after receiving vaccines/placebo and provocation compared with normal mice as determined by the quantitative real-time PCR. ^*^, *P* < 0.05 compared with L-P; ^#^, *P* < 0.05 compared with normal mice by independent *t*-test.

Fold changes of cytokine mRNAs in nasal tissues of allergic mice after treatments in comparison with the normal mice are shown in Fig 4. *IL-4*, *IL-5* and *IL-13* mRNAs in the L-nFD1 vaccinated allergic mice were lower than the placebo mice (*p* < 0.05) (Fig 4A, 4B and 4C). Among the three Th2 cytokines, only *IL-5* mRNA in the L-cCE mice was lower than the placebo mice (Fig 4B). *TNF-α* mRNAs of both vaccinated mouse groups reduced markedly compared to the placebo (*p* < 0.05) (Fig 4D). The levels of the *IL-12a* (*p35*), *IL-12b (p40)* and *IL-23 (p19)* mRNAs of L-cCE and L-nFD1 groups were lower than the L-P group (*p* < 0.05) (Fig 4E, 4F and 4G, respectively). *IFN-γ* mRNAs in the vaccinated and placebo mice were not different (Fig 4H). The vaccinated mice had significant increase in *IL-10* and *TGF-β* mRNA levels (*p* < 0.05) (Fig 4I and 4J) compared to the placebo and normal mice, while those of the L-P treated mice were significantly lower than the normal mice (*p* < 0.05) On contrary, expressions of *IL-35* gene (*ebi3*) in the L-cCE and L-nFD1 groups were significantly lower than the placebo (*p* < 0.05) (Fig 4K).

## Discussion

Asthma models of cat allergy have been developed previously [23–26] but the model of allergic rhinitis (AR) has not been established as yet as far as the literature review. Therefore in this study, the AR model to cCE was developed in mice by using the method and timeline that were modified from previously successful allergy model development [9,17,26,27]. Nasal symptom scores [27–29] were used for monitoring AR of the allergenized mice. The cCE allergenized mice had more frequent nose rubbing and sneezing, more inflammatory cell infiltration into the nasal tissues, more nasal mucus production, and higher serum specific IgE and IgG1 than the sham mice. The overall features indicate that the allergenized mice had allergy (allergic rhinitis) [30] even though the mast cell number at the tips of the allegenized mouse noses were not different from the sham mice. Usually the mouse mast cells predominate at the body surface areas that exposed to the external environment [31]. The cells are not involved only in anaphylaxis or allergy but they also mediate immune reaction (innate immunity) to foreign matters that have arrived at the respiratory tissues [32]. Irritation of the mouse nasal tissues by allergen and buffer instillation and nebulization could recruit the mast cells to the nasal tissues in, more or less, similar degree.

Multi-lamellar liposome was chosen as the vaccine delivery vehicle/adjuvant as it is non-toxic, biodegradable, and compatible with mammalian tissues. The encapsulated cargo can be protected from the host hostile environment, *e*.*g*., enzymatic degradation [33]. The antigen is released slowly from the micelles; thus reducing possibility of the antigen-mediated toxicity. Liposome is known to be a Th1 adjuvant [34]. Nevertheless, types of the induced immune response depend also on the liposome sizes [35]. The large vesicles (≥ 225 nm) were usually phagocytosed by macrophages which involved in Th1 response [36,37] while the small vesicles (≤ 155 nm) were captured by B lymphocytes [35,36]. The sizes of all liposome-entrapped vaccines in this study were above 3.5 μm; therefore, they should stimulate the Th1 response to the entrapped components. The liposome composed of the phosphatidylcholine (neutral phospholipid) and cholesterol were used successfully as the vaccine delivery vehicle and adjuvant for treatment of allergies in mouse models [9,38]. The cationic liposome have more chance of coalescing with the negatively charged host cell membrane and able to retain antigen at the site of administration with higher ability to stimulate dendritic cells than the neutral or anionic vesicles [39,40]. The cationic surfactant, *i*.*e*., DDAB, was used in an attempt to create the positive surface charges to the liposome-vaccines. The L-nFD1 and the L-P were cathodic as expected, but the L-cCE had a slightly anionic charge (-2.31 ± 0.25 mV), most possibly due the unknown components in the crude extract which are beyond the control. The concentration of nFel d 1 for each vaccine dose in this study was based on the previous intranasal liposome-adjuvanted refined major American cockroach allergen vaccine which was effective in treatment of the cockroach allergy in a mouse model [9].

Levels of serum-specific IgE, a pathogenic antibody isotype for allergy, in both vaccinated groups were reduced compared to the placebo. The role of IgG in allergy development has been controversial because of their binding affinity to different Fcγ receptors which may lead to different immune response outcomes [41–44]. In SIT, IgG can block the allergen binding to IgE on the mast cell/basophil surface and thereby inhibits the allergic responses [44–46]. In this study, both IgG1 and IgG2a rose in vaccinated allergic mice. The rise of specific IgG responses with the reduction of IgE levels has lowered the IgE:IgG1 and IgE:IgG2 ratios among the vaccinated groups in comparison to the placebo which indicates a shift of the Th2 to the Th1 response by the vaccines.

The L-cCE vaccinated mice had the highest number of the cells infiltrated into nasal tissues which might be a result of the non-target allergenic components contained in the cCE [47].

Both vaccines mediated reduction of the grade 3 mucus gland intensities which conformed to the previously finding that allergic mice had reduction of mucus production after SIT [26,45]. The *IL-4*, *IL-5* and *IL-13* in the nasal tissues of the L-nFD1 treated mice were reduced significantly while only the *IL-5* mRNA was reduced in the L-cCE group, implying the higher efficacy of the former than the latter in reducing the Th2 response. These findings are conformed to the data reported previously [9]. The reduction of the Th2 cytokine gene expressions might be a cause of mucus production inhibition in the vaccinated mice [48].

TNF-α is a pro-inflammatory cytokine released in allergic responses from mast cells and macrophages *via* IgE-dependent mechanisms [49]. It is required for allergen-specific IgE production, induction of Th2 cytokines, and expression of adhesion molecule on endothelial cells including ELAM-1, VCAM-1 and ICAM-1 which are involved in eosinophil infiltration to the site of allergic inflammation [50]. In this study, the *TNF-α* mRNA of allergic mice was reduced after vaccination, which suggests again the reduction of the allergen specific Th2 response.

Recently, IL-23 has been identified as a novel member of IL-12 family. Each molecule of this cytokine is composed of p19 subunit specific for IL-23 and IL-12p40. IL-23 is required for Th17 maintenance. The IL-23/Th17 cell axis plays a key role in development of inflammation including autoimmune diseases and allergy [51,52]. Allergic mice treated with both liposome vaccines had marked reduction of the *p19* mRNA compared to the placebo mice, indicating a propensity of allergic inflammation reduction by regulating the Th17 while promoting the regulatory T cells (see below).

IFN-γ inhibits Th2 cytokines [53–55]. By the time of vaccine efficacy analysis, the *IFN-γ* mRNAs of the vaccinated mice were not different from the placebo although they were higher than the normal mice. However, there was a marked reduction of the *IL-35* mRNAs among the vaccinated mice compared to the placebo mice. The IL-35 is a heterodimer of EBI3 and IL-12a/p35 which is produced by both regulatory T and B lymphocytes [56,57]. IL-35 is a Th1 specific immunosuppressive cytokine [56]. The *IL-12p35/ebi3* mRNAs in nasal tissues of allergic mice were reduced after treatment with the vaccines; thus making significant increases of the ratios of *IFN-γ* to *IL-35* mRNA expressions (0.46 ± 0.16 for L-cCE group and 0.51 ± 0.24 for L-nFD1 group) which were significantly higher than those of placebo group (0.07 ± 0.02) (*p* < 0.05). These data suggest that there was a trend of the Th1 up-regulation.

Apart from the production of IL-35, regulatory T cells also produce IL-10 and TGF-β which involved in an immune-regulation for allergy by suppressing effector T cell response(s), inhibiting allergen-specific IgE production, and inducing IgG4 and/or IgA production in human after SIT [58,59]. Both vaccinated mouse groups had significant increases of *IL-10* and *TGF-β* mRNAs compared to the placebo mice, indicating Treg generation after vaccination.

## Conclusions

A mouse model of allergic rhinitis model to crude cat hair extract (cCE) was developed. The cat allergen vaccines alleviated the allergic manifestations in the modeled mice by causing a shift of the pathogenic Th2 response towards the non-pathogenic Th1 and Treg responses. The liposome-adjuvanted cat allergen vaccines, particularly the L-nFel d 1, is worth testing further for clinical applications.

## Supporting Information

S1 Fig Experimental time-line of cat allergy model development (PDF)

S2 Fig Experimental time-line of vaccination, provocation and vaccine efficacy evaluation. (PDF)

S1 Table Oligonucleotide primers used for quantitative real-time PCR of monitoring cytokine gene expressions. (PDF)

S2 Table Ratios of specific IgE to IgG1 and IgE to IgG2a in sera of vaccinated and placebo allergic mice. (PDF)

## Abbreviations

AR: allergic rhinitis
cCE: cat crude hair/dander extract
DDAB: didecyldioctadecylammonium bromide
ELAM-1: endothelial leukocyte adhesion molecule-1
ICAM1: intercellular cell adhesion molecule-1
IL: interleukin
L: liposome
nFel d 1: native Fel d 1
Ni-NTA: Nickel-nitrilotriacetic acid
PAS: Periodic acid-Schiff
PDI: polydispersity index (-ices)
TGF-β: transforming growth factor-beta
TNF-α: tumor necrosis factor-alfa
VCAM1: vascular cell adhesion molecule-1

## References

[1] Chapman MD, Aalberse RC, Brown MJ, Platts-Mills TA. Monoclonal antibodies to the major feline allergen Fel d 1. II. Single step affinity purification of Fel d 1, N-terminal sequence analysis, and development of a sensitive two-site immunoassay to assess Fel d 1 exposure. J Immunol. 1988;140(3):812–8. 3276780

[2] Bunnag C, Jareoncharsri P, Tantilipikorn P, Vichyanond P, Pawankar R. Epidemiology and current status of allergic rhinitis and asthma in Thailand–ARIA Asia-Pacific Workshop report. Asian Pac J Allergy Immunol. 2009;27(1):79–86. 19548633

[3] Bollinger ME, Eggleston PA, Wood RA. Cat antigens in homes with and without cats may induce allergic symptoms. J Allergy Clin Immunol. 1997;97:907–14. 8655885 10.1016/s0091-6749(96)80064-9

[4] Phipatanakul W. Animal allergens and their control. Curr Allergy Asthm Rep. 2001;1:461–5. 11892073 10.1007/s11882-001-0034-2

[5] de Groot H, van Swieten P, van Leeuwen J, Lind P, Aalberse RC. Monoclonal antibodies to the major feline allergen Fel d 1. I. Serologic and biologic activity of affinity-purified Fel d 1 and of Fel d 1-depleted extract. J Allergy Clin Immunol. 1988;82(5):778–86. 2461402 10.1016/0091-6749(88)90079-6

[6] Larché M, Akdis CA, Valenta R. Immunological mechanisms of allergen-specific immunotherapy. Nat Rev Immunol. 2006;6:761–71. 16998509 10.1038/nri1934

[7] Olivieri M, Mohaddes-Zadeh MR, Talamini G, Lampronti G, Lo-Cascio V. Local nasal immunotherapy and bronchial hyperreactivity in seasonal allergic rhinitis: an observational pilot study. J Investig Allergol Clin Immunol. 2000;10(5):300–4. 11108443

[8] Alving CR. Liposomes as carriers of antigens and adjuvants. J Immunol Methods. 1991;140(1):1–13. 1712030 10.1016/0022-1759(91)90120-5

[9] Meechan P, Tungtrongchitr A, Chaisri U, Maklon K, Indrawattana N, Chaicumpa W, et al Intranasal, liposome-adjuvanted cockroach allergy vaccines made of refined major allergen and whole-body extract of *Periplaneta americana*. Int Arch Allergy Immunol. 2013;161(4):351–62. 10.1159/000348314 . 23689057

[10] Valenta R. The future of antigen-specific immunotherapy of allergy. Nat Rev Immunol. 2002;2(6):446–53. 12093011 10.1038/nri824

[11] Ball T, Linhart B, Sonneck K, Blatt K, Herrmann H, Valent P, et al Reducing allergenicity by altering allergen fold: a mosaic protein of Phl p 1 for allergy vaccination. Allergy. 2009;64(4):569–80. 10.1111/j.1398-9995.2008.01910.x 19243361

[12] Valenta R, Campana R, Marth K, van Hage M. Allergen-specific immunotherapy: from therapeutic vaccines to prophylactic approaches. J Intern Med. 2012;272(2):144–57. 10.1111/j.1365-2796.2012.02556.x 22640224 PMC4573524

[13] Rogers BL, Morgenstern JP, Garman RD, Bond JF, Kuo MC. Recombinant Fel d 1: expression, purification, IgE binding and reaction with cat-allergic human T cells. Mol Immunol. 1993;30(6):559–68. 8487777 10.1016/0161-5890(93)90030-f

[14] Badiee A, Jaafari MR, Khamesipour A, Samiei A, Soroush D, Kheiri MT, et al The role of liposome charge on immune response generated in BALB/c mice immunized with recombinant major surface glycoprotein of *Leishmania* (rgp63). Exp Parasitol. 2009;121(4):362–9. 10.1016/j.exppara.2008.12.015 19211022

[15] Herbert DR, Hölscher C, Mohrs M, Arendse B, Schwegmann A, Radwanska M, et al Alternative macrophage activation is essential for survival during schistosomiasis and down modulates T helper 1 responses and immunopathology. Immunity. 2004;20(5):623–35. 15142530 10.1016/s1074-7613(04)00107-4

[16] Ryang YS, Yang EJ, Kim JL, Lee KJ, Sung HJ, Kim JB, Kim IS. Immune response and inhibitory effect of ketotifen on the BALB/c and C3H/HeN mice infected with *Echinostoma hortense*. Parasitol Res. 2007;101(4):1103–10. 17618462 10.1007/s00436-007-0591-y

[17] Sookrung N, Indrawattana N, Tungtrongchitr A, Karuhassuwan C, Chaisri U, Chaicumpa W. A murine model of allergy caused by American cockroach (CR), *Periplaneta americana*. Asian Pac J Allergy Immunol. 2008;26(2–3):143–9. 19054933

[18] Czarneski J, Lin YC, Chong S, McCarthy B, Fernandes H, Parker G, et al Studies in NZB IL-10 knockout mice of the requirement of IL-10 for progression of B-cell lymphoma. Leukemia. 2004;18(3):597–606. 14712288 10.1038/sj.leu.2403244

[19] Schuetze N, Schoeneberger S, Mueller U, Freudenberg MA, Alber G, Straubinger RK. IL-12 family members: differential kinetics of their TLR4-mediated induction by *Salmonella enteritidis* and the impact of IL-10 in bone marrow-derived macrophages. Int Immunol. 2005;17(5):649–59. 15837713 10.1093/intimm/dxh247

[20] Overbergh L, Valckx D, Waer M, Mathieu C. Quantification of murine cytokine mRNAs using real-time quantitative reverse transcriptase PCR. Cytokine. 1999;11(4):305–12. 10328870 10.1006/cyto.1998.0426

[21] Lehmann J., Bellmann S., Werner C., Schroder R., Schutze N., Alber G. IL-12p40-dependent agonistic effects on the development of protective innate and adaptive immunity against *Salmonella enteritidis*. J Immunol. 2001;167(9):5304–15. 11673546 10.4049/jimmunol.167.9.5304

[22] Boeuf P, Vigan-Womas I, Jublot D, Loizon S, Barale JC, Akanmori DB, et al CyProQuant-PCR: a real-time RT-PCR technique for profiling human cytokines, based on external RNA standards, readily automatable for clinical use. BMC Immunol. 2005;6:5 15748278 10.1186/1471-2172-6-5 PMC555737

[23] Briner TJ, Kuo MC, Keating KM, Rogers BL, Greensein JL. Peripheral T-cell tolerance induced in naïve and primed mice by subcutaneous injection of peptides from the major cat allergen Fel d 1. Proc Natl Acad Sci USA. 1993;90(16):7608–12. 8356062 10.1073/pnas.90.16.7608 PMC47191

[24] Treter S, Lugman M. Antigen-specific T cell tolerance down-regulates mast cell responses *in vivo*. Cell Immunol. 2000;206(2):116–24. 11161443 10.1006/cimm.2000.1739

[25] Martinez-Gómez JM, Johansen P, Erdmann I, Senti G, Crameri R, Kündig TM. Intralymphatic injections as a new administration route for allergen-specific immunotherapy. Int Arch Allergy Immunol. 2009;150(1):59–65. 10.1159/000210381 19339803

[26] Grundström J, Neimert-Andersson T, Kemi C, Nilsson OB, Saarne T, Andersson M, et al Covalent coupling of vitamin D3 to the major cat allergen Fel d 1 improves the effects of allergen-specific immunotherapy in a mouse model of cat allergy. Int Arch Allergy Immunol. 2012;157(2):136–46. 10.1159/000327546 21985799

[27] Mo JH, Kang EK, Quan SH, Rhee CS, Lee CH, Kim DY. Anti-tumor necrosis factor-alpha treatment reduces allergic responses in an allergic rhinitis mouse model. Allergy. 2011;66(2):279–86. 10.1111/j.1398-9995.2010.02476.x 21208219

[28] Brimnes J, Kildsgaard J, Jacobi H, Lund K. Sublingual immunotherapy reduces allergic symptoms in a mouse model of rhinitis. Clin Exp Allergy. 2006;37(4):488–97. 17430344 10.1111/j.1365-2222.2006.02624.x

[29] Wang M, Zhang W, Shang J, Yang J, Zhang J, Bachert C. Immunomodulatory effects of IL-23 and IL-17 in a mouse model of allergic rhinitis. Clin Exp Allergy. 2013;43(8):956–66. 10.1111/cea.12123 23889249

[30] Baraniuk JN. Mechanisms of allergic rhinitis. Curr Allergy Asthma Rep. 2001;1(3):207–17. 11892038 10.1007/s11882-001-0007-5

[31] Galli SJ, Borregaard N, Wynn TA. Phenotypic and functional plasticity of cells of innate immunity: macrophages, mast cells and neutrophils. Nat Immunol. 2011;12(11):1035–44. 10.1038/ni.2109 22012443 PMC3412172

[32] Ebmeyer J, Ebmeyer U, Pak K, Sudhoff H, Broide D, Ryan AF, Wasserman S. Reconstitution of the mast cell population in W/W^v^ mice. Otol Neurotol 2010;31(1): 42–47. 10.1097/MAO.0b013e3181b4e3e3 19752767 PMC2796298

[33] Akbarzadeh A, Rezaei-Sadabady R, Davaran S, Joo SW, Zarghami N, Hanifehpour Y, et al Liposome: classification, preparation, and applications. Nanoscale Res Lett. 2013;8(1):102 10.1186/1556-276X-8-102 23432972 PMC3599573

[34] Gregoriadis G. Immunological adjuvants: a role for liposomes. Immunol Today. 1990;11(3):89–97. 2186746 10.1016/0167-5699(90)90034-7

[35] Brewer JM, Tetley L, Richmond J, Liew FY, Alexander J. Lipid vesicle size determines the Th1 or Th2 response to entrapped antigen. J Immunol. 1998;161(8):4000–7. 9780169

[36] Del Monte P, Szoka FC Jr. Effect of liposome encapsulation on antigen presentation *in vitro*. Comparison of presentation by peritoneal macrophages and B cell tumors. J Immunol. 1989;142(5):1437–43. 2465339

[37] Desmedt M, Rottiers P, Dooms H, Fiers W, Grooten J. Macrophages induce cellular immunity by activating Th1 cell responses and suppressing Th2 cell responses. J Immunol. 1998;160(11):5300–8. 9605128

[38] Basomba A, Tabar AI, de Rojas DH, Garcia BE, Alamar R, Olaguibel JM, et al Allergen vaccination with a liposome-encapsulated extract of *Dermatophagoides pteronyssinus*: a randomized, double-blind, placebo-controlled trial in asthmatic patients. J Allergy Clin Immunol. 2002;109(6):943–8. 12063522 10.1067/mai.2002.124465

[39] Vangasseri DP, Cui Z, Chen W, Hokey DA, Falo LD Jr, Huang L. Immunostimulation of dendritic cells by cationic liposomes. Mol Membr Biol. 2006;23(5):385–95. 17060156 10.1080/09687860600790537

[40] Henriksen-Lacey M, Christensen D, Bramwell VW, Lindenstrøm T, Agger EM, Andersen P, et al Liposomal cationic charge and antigen adsorption are important properties for the efficient deposition of antigen at the injection site and ability of the vaccine to induce a CMI response. J Control Release. 2010;145(2):102–8. 10.1016/j.jconrel.2010.03.027 20381556

[41] Samsom JN, van Berkel LA, van Helvoort JMLM, Unger WWJ, Jansen W, Thepen T, et al FcγRIIB regulates nasal and oral tolerance: a role of dendritic cells. J Immunol. 2005;174(9):5279–87. 15843524 10.4049/jimmunol.174.9.5279

[42] Ishikawa Y, Kobayashi K, Yamamoto M, Nakata K, Takagawa T, Funada Y, et al Antigen-specific IgG ameliorates allergic airway inflammatory *via* Fcγ receptor IIB on dendritic cells. Respir Res. 2011;12:42 10.1186/1465-9921-12-42 21477339 PMC3079623

[43] Bruhns P. Properties of mouse and human IgG receptors and their contribution to disease models. Blood. 2012;119(24):5640–9. 10.1182/blood-2012-01-380121 22535666

[44] Williams JW, Tjota MY, Sperling AI. The contribution of allergen-specific IgG to the development of Th2-mediated airway inflammation. J Allergy (Cairo). 2012;2012:236075 10.1155/2012/236075 23150737 PMC3485540

[45] Neimert-Andersson T, Thunberg S, Swedin L, Wiedermann U, Jacobsson-Ekman G, Dahlén SE, et al Carbohydrate-based particles reduce allergic inflammation in a mouse model for cat allergy. Allergy. 2008;63(5):518–26. 10.1111/j.1398-9995.2008.01644.x 18394125

[46] Saarne T, Neimert-Andersson T, Grönlund H, Jutel M, Gafvelin G, van Hage M. Treatment with a Fel d 1 hypoallergen reduces allergic responses in a mouse model of cat allergy. Allergy. 2011;66(2):255–63. 10.1111/j.1398-9995.2010.02468.x 20804464

[47] Focke M, Swoboda I, Marth K, Valenta R. Developments in allergen-specific immunotherapy: from allergen extracts to allergy vaccines bypassing allergen-specific immunoglobulin E and T cell reactivity. Clin Exp Allergy. 2010;40:385–97. 10.1111/j.1365-2222.2009.03443.x 20210812

[48] Cohn L, Homer RJ, MacLeod H, Mohrs M, Brombacher F, Bottomly K. Th2-induced airway mucus production is dependent on IL-4Rα, but not on eosinophils. J Immunol 1999;162:6178–83. 10229862

[49] Thomas PS. Tumor necrosis factor-alpha: the role of this multifunctional cytokine in asthma. Immunol Cell Biol. 2001;79(2):132–40. 11264706 10.1046/j.1440-1711.2001.00980.x

[50] Iwasaki M, Saito K, Takemura M, Sekikawa K, Fujii H, Yamada Y, et al TNF-alpha contributes to the development of allergic rhinitis in mice. J Allergy Clin Immunol. 2003;122(1):134–40. 12847490 10.1067/mai.2003.1554

[51] McGeachy MJ, Bak-Jansen KS, Chen Y, Tato CM, Blumenschein W, McClanahan T, et al TGF-beta and IL-6 drive the production of IL-17 and IL-10 by T cells and restrain T(H)17 cell-mediated pathology. Nat Immunol. 2007;8:1390–7. 17994024 10.1038/ni1539

[52] Astry B, Venkatesha SH, Moudgil K. Involvement of the IL-23/Th17 axis and the Th17/Treg balance in the pathogenesis and control of autoimmune arthritis. Cytokine. 2015;74:54–61. 10.1016/j.cyto.2014.11.020 25595306 PMC4457562

[53] Maggi E, Parronchi P, Manetti R, Simonelli C, Piccinni MP, Rugiu FS, et al Reciprocal regulatory effects of IFN-gamma and IL-4 on the *in vitro* development of human Th1 and Th2 clones. J Immunol. 1992;148(7):2142–7. 1532000

[54] Durham SR, Ying S, Varney VA, Jacobson MR, Sudderick RM, Mackay IS, et al Grass pollen immunotherapy inhibits allergen-induced infiltration of CD4+ T lymphocytes and eosinophils in the nasal mucosa and increases the number of cells expressing messenger RNA for interferon-gamma. J Allergy Clin Immunol. 1996;97:1356–65. 8648033 10.1016/s0091-6749(96)70205-1

[55] Wilson DR, Nouri-Aria KT, Walker SM, Pajno GB, O’Brien F, Jacobson MR, et al Grass pollen immunotherapy: symptomatic improvement correlates with reductions in eosinophils and IL-5 mRNA expression in the nasal mucosa during the pollen season. J Allergy Clin Immunol. 2001;107:971–6. 11398073 10.1067/mai.2001.115483

[56] Shen P, Roch T, Lampropoulou V, O'Connor RA, Stervbo U, Hilgenberg E, et al IL-35-producing B cells are critical regulators of immunity during autoimmune and infectious disease. Nature. 2014;507(7492):366–70. 10.1038/nature12979 24572363 PMC4260166

[57] Collison LW, Chaturvedi V, Henderson AL, Giacomin PR, Guy C, Bankoti J, et al IL-35-mediated induction of a potent regulatory T cell population. Nat Immunol. 2010;11(12):1093–101. 10.1038/ni.1952 20953201 PMC3008395

[58] Jutel M, Akdis M, Budak F, Aebischer-Casaulta C, Wrzys M, Blaser K, et al IL-10 and TGF-β cooperate in the regulatory T cell response to mucosal allergens in normal immunity and specific immunotherapy. Eur J Immunol. 2003;33:1205–14. 12731045 10.1002/eji.200322919

[59] Akdis M, Akdis CA. Mechanisms of allergen-specific immunotherapy: multiple suppressor factors at work in immune tolerance to allergens. J Allergy Clin Immunol. 2014;133:621–31. 10.1016/j.jaci.2013.12.1088 24581429

