# Supplementary material for: Mouse Model of Cat Allergic Rhinitis and Intranasal Liposome-Adjuvanted Refined Fel d 1 Vaccine
Source: PLoS One. 2016 Mar 8;11(3):e0150463. doi: 10.1371/journal.pone.0150463 (PMC4783078; doi:10.1371/journal.pone.0150463)
Supplement: S1 Fig — (PDF) [file pone.0150463.s001.pdf]

Supporting Information

Allergic Rhinitis Model of Cat Allergy and Intranasal Liposome-Adjuvanted Refined Fel d 1 Vaccine

Natt Tasaniyananda[1,2], Urai Chaisri[3], Anchalee Tungtrongchitr[2], Wanpen Chaicumpa[2], Nitat Sookrung[4*]

[1]Graduate Program in Immunology, Department of Immunology, [2]Laboratory for Research and Technology Development, Department of Parasitology and [4]Office for Research and Development, Faculty of Medicine Siriraj Hospital, Mahidol University, Bangkok 10700, Thailand; [3]Department of Tropical Pathology, Faculty of Tropical Medicine, Mahidol University, Bangkok 10400, Thailand

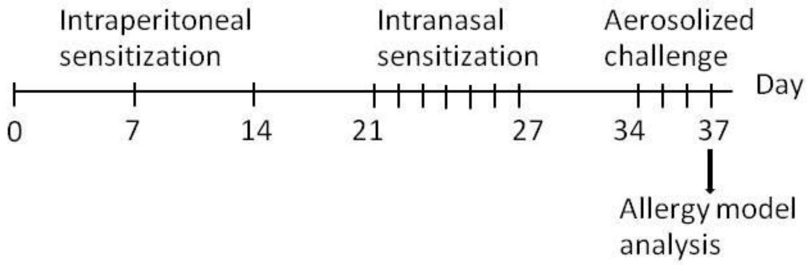

S1 Fig. Experimental time-line of cat allergy model development.
