# Supplementary material for: Mouse Model of Cat Allergic Rhinitis and Intranasal Liposome-Adjuvanted Refined Fel d 1 Vaccine
Source: PLoS One. 2016 Mar 8;11(3):e0150463. doi: 10.1371/journal.pone.0150463 (PMC4783078; doi:10.1371/journal.pone.0150463)
Supplement: S2 Fig — (PDF) [file pone.0150463.s002.pdf]

# Supporting Information

## Allergic Rhinitis Model of Cat Allergy and Intranasal Liposome-Adjuvanted Refined Fel d 1 Vaccine

**Natt Tasaniyananda[1,2], Urai Chaisri[3], Anchalee Tungtrongchitr[2], Wanpen Chaicumpa[2], Nitat Sookrung[4*]**

[1]Graduate Program in Immunology, Department of Immunology, [2]Laboratory for Research and Technology Development, Department of Parasitology and [4]Office for Research and Development, Faculty of Medicine Siriraj Hospital, Mahidol University, Bangkok 10700, Thailand; [3]Department of Tropical Pathology, Faculty of Tropical Medicine, Mahidol University, Bangkok 10400, Thailand

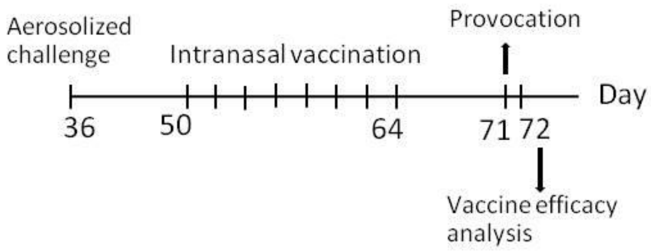


**S2 Fig.** Experimental time-line of vaccination, provocation and vaccine efficacy evaluation.
