# Supplementary material for: Mouse Model of Cat Allergic Rhinitis and Intranasal Liposome-Adjuvanted Refined Fel d 1 Vaccine
Source: PLoS One. 2016 Mar 8;11(3):e0150463. doi: 10.1371/journal.pone.0150463 (PMC4783078; doi:10.1371/journal.pone.0150463)
Supplement: S1 Table — (PDF) [file pone.0150463.s003.pdf]

Supporting Information

Allergic Rhinitis Model of Cat Allergy and Intranasal Liposome-Adjuvanted Refined Fel d 1 Vaccine

Natt Tasaniyananda[1,2], Urai Chaisri[3], Anchalee Tungtrongchitr[2], Wanpen Chaicumpa[2], Nitat Sookrung[4*]

[1]Graduate Program in Immunology, Department of Immunology, [2]Laboratory for Research and Technology Development, Department of Parasitology and [4]Office for Research and Development, Faculty of Medicine Siriraj Hospital, Mahidol University, Bangkok 10700, Thailand; [3]Department of Tropical Pathology, Faculty of Tropical Medicine, Mahidol University, Bangkok 10400, Thailand

S1 Table. Oligonucleotide Primers Used in Quantitative Real-time PCR for Monitoring Cytokine Gene Expressions.

**Gene**	**Primer**	**Size of PCR product (bp)**
IL-4	F: 5’-TCGGCATTTTGAACGAGGTC-3’<br>R:5’-GAAAAGCCCGAAAGAGTCTC-3’	218
IL-5	F: 5’-ATGATCGTGCCTCTGTGCCTGGAGC-3’<br>R:5’-CTGTTTTTCCTGGAGTAAACTGGGG-3’	242
IL-13	F: 5’-CGCTGGCGGGTTCTGTGTAG-3’<br>R:5’-GAGGCTGGAGACCGTAGTGGG-3’	121
TNF-α	F: 5’-CATCTTCTCAAAATTCGAGTGACAA-3’<br>R:5’-TGGGAGTAGACAAGGTACAACCC-3’	174
IL-12a (p35)	F: 5’- CCACCCTTGCCCTCCTAAAC-3’<br>R:5’- GTTTTTCTCTGGCCGTCTTCA -3’	132
IL-12b (p40)	F: 5’-GGAAGCACGGCAGCAGAATA-3’<br>R:5’-AACTTGAGGGAGAAGTAGGAATGG-3’	180
IL-23 (p19)	F: 5’-TGCTGGATTGCAGAGCAGTAA-3’<br>R:5’-GCATGCAGAGTTCCGAGAGA-3’	121
IFN-γ	F: 5’-AACGCTACACACTGCATCTTGG-3’<br>R:5’-GACTTCAAAGAGTCTGAGG-3’	237
IL-35 (ebi3)	F: 5’-CAATGCCATGCTTCTCGGTAT-3’<br>R:5’-GGACGTGGATCTGGTGGAGTT-3’	84
IL-10	F: 5’-CGGGAAGACAATAACTG-3’<br>R:5’-CATTTCCGATAAGGCTTGG-3’	191
TGF-β	F: 5’-CAAGGGCTACCATGCCAACT-3’<br>R:5’-AGGGCCAGGACCTTGCTG-3’	84
β-actin	F: 5’-GGCCAACCGTGAAAAGATGA-3’<br>R:5’-CACGCTCGGTCAGGATCTTC-3’	251
