# Supplementary material for: Mouse Model of Cat Allergic Rhinitis and Intranasal Liposome-Adjuvanted Refined Fel d 1 Vaccine
Source: PLoS One. 2016 Mar 8;11(3):e0150463. doi: 10.1371/journal.pone.0150463 (PMC4783078; doi:10.1371/journal.pone.0150463)
Supplement: S2 Table — (PDF) [file pone.0150463.s004.pdf]

# Supporting Information

# Allergic Rhinitis Model of Cat Allergy and Intranasal Liposome-Adjuvanted Refined Fel d 1 Vaccine

**Natt Tasaniyananda[1,2], Urai Chaisri[3], Anchalee Tungtrongchitr[2], Wanpen Chaicumpa[2], Nitat Sookrung[4*]**

[1]Graduate Program in Immunology, Department of Immunology, [2]Laboratory for Research and Technology Development, Department of Parasitology and [4]Office for Research and Development, Faculty of Medicine Siriraj Hospital, Mahidol University, Bangkok 10700, Thailand; [3]Department of Tropical Pathology, Faculty of Tropical Medicine, Mahidol University, Bangkok 10400, Thailand

**S2 Table. Ratios of Specific IgE to IgG1 and IgE to IgG2a in Sera of Vaccinated and Placebo Allergic Mice.**

| **Group of mice** | **IgE:IgG1** | **IgE:IgG2a** |
|---|---|---|
| L-P | 0.314 ± 0.197 | 2.375 ± 1.373 |
| L-cCE | 0.05 ± 0.037* | 0.104 ± 0.051* |
| L-nFD1 | 0.145 ± 0.081* | 0.635 ± 0.713* |

* $p < 0.05$ compared with L-P by independent *t*-test. $n = 5$ per group.
